# Enhancement of paclitaxel production by *Neopestalotiopsis vitis* via optimization of growth conditions

**DOI:** 10.1371/journal.pone.0309325

**Published:** 2024-10-15

**Authors:** Hamzeh Rezazadeh, Faezeh Ghanati, Mercedes Bonfill, Fatemeh Nasibi, Narjes Mohammadi Ballakuti

**Affiliations:** 1 Department of Plant Biology, Faculty of Biological Science, Tarbiat Modares University, Tehran, Iran; 2 Department of Biology, Healthcare and the Environment, Faculty of Pharmacy and Food Sciences, University of Barcelona, Barcelona, Spain; 3 Department of Biology, Faculty of Sciences, Shahid Bahonar University of Kerman, Kerman, Iran; Hainan University, CHINA

## Abstract

Accessibility of paclitaxel and other taxoids from natural resources is restricted. Endophytic fungi are novel, rapidly growing resources for producing these compounds. *Neopestalotiopsis vitis (N*. *vitis)* has been recently isolated from *Corylus avellana*, and its ability to produce a variety of taxoids has been detected and confirmed by analytical methods. Simultaneous growth and high production of taxoids by application of different sorts and concentrations of carbon and nitrogen were targeted in the present research. These criteria were assessed in different acidities (pH 4.0–7.0), carbon sources (sucrose, fructose, glucose, mannitol, sorbitol, and malt extract), and nitrogen forms (urea, ammonium nitrate, potassium nitrate, ammonium phosphate, and ammonium sulfate) by testing one parameter at a time approach. The first analysis introduced pH 7.0 as the best acidity of the medium for *N*. *vitis*, where the highest paclitaxel yield was generated. Further analysis introduced 3% Malt extract as the best carbon-providing medium. In the next step, the effects of nitrogen forms on the growth rate, paclitaxel yield, alkaloids, and amino acid contents were evaluated. Based on the results of this experiment, 5 mM ammonium sulfate was selected as the best nitrogen source to obtain the maximum biomass and paclitaxel yield. Overall, the results introduce a medium containing 3% (w/v) malt extract and 5 mM ammonium sulfate at pH 7.0 as the best medium in which *N*. *vitis* produces the highest paclitaxel yield coincident with rapid and sustainable growth. The findings pave the way for industrial manufacturing of taxoids.

## Introduction

Paclitaxel (taxol) is one of the most effective cancer drugs compared with other anti-tumor drugs. In the past, it was mainly used to treat breast and ovarian cancers, as well as leukemia [1], lymph nodes [2], lung [3], colon [4], head [5] and neck cancers [6], and cervical and prostate cancer [7]. Paclitaxel and other taxoids profoundly arrest the cell cycle of cancerous cells during the G_2_/M phase, eventually resulting in their death [8]. This drug has been basically derived from *Taxus* sp., which is a very slow-growing plant species.

Paclitaxel has a novel terpene-based alkaloid structure and is produced during a long biosynthesis pathway using almost twenty different enzymes. Moreover, the strategy of semi-synthetic paclitaxel and other taxoids using *Taxus*-derived precursors is time-consuming and too expensive [9]. Moreover, the limited availability of the plant and greater vulnerability to unpredicted changes in ecological and environmental conditions, low selectivity over unwanted byproducts, heterogeneity, the risk of epigenetic and mutational changes, and low reproducibility make this source challenging, resulting in a demand for further studies to find new resources for this drug.

Extensive research has been started in the last few decades introducing endophytic fungi of *Taxus* and *Corylus avellana* as alternative natural resources for different taxoids. Endophytic fungi are a group of microorganisms that reside within the tissues of plants with a mutualistic, not pathogenic relationship with the host plant and produce myriads of compounds known for their antibiotic [10], antifungal [11], antibacterial [12], and other desirable properties [13]. *Aspergillus fumigatus*, *Alternaria tenuissima*, *Alternaria alternata*, *Cladosporium variabile*, *Penicillium polonicum*, *Fusarium tricinctum*, and *Neopestalotiopsis vitis* are some instances of taxoids-producing fungi [14–16]. Research on these fungi can contribute to process improvement and developing essential compounds in diverse industries while serving as reliable, eco-friendly valuable resources [17]. Nonetheless, this approach needs to tackle challenges related to enhancing the efficiency of fungi in producing the desired compounds. The augmentation of the target compounds can be achieved through diverse techniques, including genetic engineering [18], elicitation, and feeding [19].

Genetic engineering is a promising strategy for the production of secondary metabolites. Recent studies on genetic manipulation of endophytic fungi have mainly focused on overexpression [20], targeted gene disruption by CRISPR/Cas9 technology [21], deletion of the essential gene of one biosynthetic pathway and activation of other silent genes [20], and silencing of rate-limiting genes and transcriptional factors [22]. Given the scarcity of genomic information and experimental difficulties, most secondary metabolite biosynthetic pathways, particularly for taxoids, still need to be discovered in endophytic fungi. Molecular research on taxanes-producing endophytic fungi has focused more on identifying and sequencing paclitaxel-producing genes [23].

Researchers examined the transcriptomes of a *Taxus-*endophytic strain of *Aspergillus* and a mutant strain that produces higher levels of taxanes. They analyzed the changes in gene expression between the two strains to better understand the underlying mechanisms that drive taxane production [24]. It was interesting to discover that even though the paclitaxel yield was higher, only the genes related to the mevalonate pathway were upregulated. The downstream genes in the taxane production pathway were not expressed. However, a major concern in the genetic manipulation of endophytes is their genetic instability [22]. Downregulation of taxanes genes upon sub-culturing of the endophytic fungi has been frequently reported. Therefore, it is essential to establish growth conditions that maintain the stability of these fungi during the production of the target compounds, ensuring successful scalability.

Using elicitors derived from phytopathogens has proven to be a quite effective strategy to increase the yield of specific metabolites [25]. Chemical elicitors like salicylic acid, sodium acetate, and serine have been commonly used to enhance the paclitaxel yield of endophytic fungi [26]. The effectiveness of elicitors is based on the hypothesis that in plant cells under stress situations, carbon shifts from the biomass production to the biosynthesis of secondary compounds as a defensive strategy. However, it should be remembered that the biosynthesis of secondary metabolites in organisms involves intricate pathways, connecting both primary and secondary metabolism. The availability and balance of essential nutrients directly influence primary metabolites and biomass, which in turn impacts the synthesis of secondary compounds in organisms [27]. The correlation between the biosynthesis of primary and secondary metabolites is fundamental for most organisms [28]. Nevertheless, the critical precursors for growth and secondary metabolites e.g. taxoids and paclitaxel are primary metabolites, requiring essential elements to be synthesized.

Current models for enhancing secondary metabolites are based on the fact that the constitutive production of secondary metabolites is coordinated with growth. Changing of cultivation method and modifying the nutrient content of the culture media is a promising technique for the simultaneously optimizing biomass production and the production of pharmaceutical secondary metabolites [29–31].

Recently, paclitaxel production has witnessed significant advancements, primarily driven by the adoption of various fermentation modes by microorganisms [32]. Fermentation strategies such as batch and semi-continuous fermentation [33], solid‐state fermentation [34], and immobilization technique [35] have been recently employed in the production of paclitaxel. However, the commercial application of any of these strategies requires the presence of essential factors such as optimal conditions of culture media. In addition, research on endophytic fungi for the production of taxoids has revealed issues regarding the instability of strains and their limited production of secondary metabolites. Utilizing an optimized culture medium is a significant stride toward improving the fermentation process and increasing the yield of desired compounds in microorganisms [36]. Therefore, conducting parallel research into factors affecting culture media and the stability of endophytic fungi could represent a significant step forward in the efficiency of beneficial metabolites and commercial purposes.

*Neopestalotiopsis vitis* is an endophyte fungus that has been recently isolated from *Corylus avellana*, and its ability to produce taxoids has been confirmed by HPLC and LC-MS analysis [37]. Previous studies have shown that the fungus overgrows in a short time on a general culture medium such as potato dextrose agar (PDA). The present study was undertaken to confirm the maintenance of taxane production potential by this fungus after a couple of subcultures and the improvement of this ability by manipulating the medium through acidity, different carbon, and nitrogen sources. The aim also included the establishment of a medium where sustainable growth is coincidently achieved with continuous taxane production.

## Martials and methods

### Chemicals

Unless otherwise stated, all chemicals were purchased from Merck (Germany). Genuine standards of taxanes were purchased from Sigma-Aldrich and ChromaDex (USA).

### Initiation of fungus suspension cultures

*Neopestalotiopsis vitis* (Accession number: MW296847) which has been isolated from hazel was used [37]. From the time of its isolation and purification, the fungus has been cultured in potato dextrose agar (PDA) (Q-Lab Corporation), at pH 5.5, 25°C ± 2, in darkness. The cultures were renewed every 10 days. The fungus’s taxane production capacity was regularly verified using HPLC and LS-MS analysis. Suspension cultures were established by immersing 0.2 g fresh mycelia into 30 mL of 3.9% potato dextrose broth (PDB) (Q-Lab Corporation) at pH 5.5. The cultures were incubated in darkness on reciprocal shakers (110 rpm), 25 ± 2°C, and were renewed every 7 days. After almost 25 sub-cultures a line of the fungus with a stable growth rate was obtained.

### Extraction and determination of taxoids

In the extraction process, the samples were homogenized in absolute MeOH (10: 1 v/w), left overnight at 25°C, and then centrifuged at 8000 ×g for 20 minutes to separate the phases. The supernatant was subsequently air-dried. A mixture of CH_2_Cl_2_ and distilled water (1:2) was added, vigorously shaken, and allowed to separate taxanes in the dichloromethane phase. The dichloromethane phase was then separated and air-dried once more. The residue was dissolved in 100 μL of MeOH, filtered through a 0.2 μm filter, and injected into a High-Performance Liquid Chromatography (HPLC) system (Waters, e2695, USA).

The HPLC system was equipped with a C18 column (Perfectsil Target ODS3, 5 μm, 250 × 4.6 mm, MZ-Analysentechnik, Mainz, Germany) and a Detector 2489 UV-Vis. The mobile phase consisted of water (containing 0.1% formic acid) and methanol, with elution performed at a flow rate of 0.8 mL min^−1^. The elution of taxanes was conducted in a gradient mode, comprising a linear gradient of 40–78% methanol over 0–30 minutes, followed by an isocratic elution with 78% methanol for 10 minutes, then decreased to 40% methanol for 5 minutes (a total run time of 45 min). Identification and quantification of taxanes were based on the retention time and peak area of genuine taxane standards (ChromaDex, USA), with each peak confirmed by injecting its corresponding standard for reassurance.

### Quantitation of total alkaloid content

Extraction and quantitation of alkaloids were conducted using the method of Yubin with modifications [38]. In brief, the samples were homogenized in 96% ethanol (EtOH), shaken overnight at 110 rpm, followed by centrifugation at 10000 × g, 10 min. The supernatant was heated for 60 min at 80°C and dried at room temperature. Subsequently, 1.0 M H_2_SO_4_ was added to the residue and subsequently extracted with diethyl ether (Et_2_O). The inorganic remained phase was made alkaline by adding 25% ammonium hydroxide (pH 9–12) and then extracted with Et_2_O. All etheric phases were concentrated by drying and resolving in Et_2_O, and their absorbance was measured at 254 nm in a double-beam spectrophotometer (GBC, Cintra 6, Australia).

### Optimization of pH, carbon and nitrogen sources

Detection of the best pH, carbon, and nitrogen sources for the growth and taxoid production by *N*. *vitis* was done stepwise as follows. In all steps, the cultures were grown in darkness on reciprocal shakers (110 rpm), 25 ± 2°C. To assess the effect of medium acidity on mycelial growth, the PDB medium with different pH values of 4.0, 5.0, 6.0, and 7.0 was prepared. Then, the fungus was transferred into these media and was allowed to grow for one week.

Based on the result of this experiment, pH 7 exhibited the most positive impact on growth rate and paclitaxel content. Therefore, the following experiments for evaluating carbon and nitrogen source types were conducted using pH 7.0. The primary concentrations of 3% (w/v) for carbon and 2.5 mM for nitrogen sources were selected based on preliminary studies and available literature [39, 40]. The effects of different carbon sources were evaluated by culturing *N*. *vitis* in 3% malt extract, sucrose, sorbitol, glucose, fructose, and mannitol for one week. Based on the results of this experiment, malt extract media was selected as the best carbon source. Therefore, the effects of different nitrogen types, including (NH_4_)_2_HPO_4_, CH_4_N_2_O, NH_4_NO_3_, (NH_4_)_2_SO_4_, and KNO_3_ on the growth rate and paclitaxel content of *N*. *vitis*, were investigated in the media containing 3% malt extract and 2.5 mM of each above-mentioned nitrogen salts (ca. 0.04%, 0.02%, 0.02%, 0.03%, and 0.03% w/v, respectively). The results of these experiments introduced ammonium sulfate as the best nitrogen source. In the third group of experiments, the effects of different concentrations of malt extract (1%, 3%, and 5%) and ammonium sulfate (1.25, 2.5, and 5 mM) on the growth and taxane production of *N*. *vitis* were evaluated for one week.

The mycelia were harvested and washed thoroughly under reduced pressure, weighed, frozen with liquid N_2_, and kept at -80 ºC for further analysis.

### Amino acids content

The samples were homogenized in 80% EtOH to determine amino acids and centrifuged at 10,000 × g for 10 min. The supernatant was isolated and dried under filtered air. The residue was dissolved in 50 μL of distilled water and agitated for 5 min at 4000 ×g. Then, 10 μL of Ortho-phthalaldehyde (OPA), 20 μL of borate buffer, and 5 μL of 0.5 M HCl were added and centrifuged at 15000 ×g for 5 min. The supernatant was injected into the HPLC system with fluorimetric detector FLD HP 1100 and using precolumn derivatization with OPA (900 μL MeOH, 100 μL of 0.1 M borate buffer, and 10 μL of β-Mercaptoethanol. Separation was carried out with a Zorbax Exlipse AAA column (4.6 × 150 mm, 3.5-μm particle size; Agilent Technologies, USA). Solvent A was a mixture of sodium phosphate buffer (25 mM, pH 7.2) and tetrahydrofuran (95:5). Solvent B was a mixture of solvent A, MeCN, and MeOH (50:35:15). A gradient composed of 0–0.6 min 10% B, 0.6–9 min 50% B, 9–48 min 60% B, 48-51min 100% B, 51–56 min 100% B, 56–57 min 10% B, 57–59 min 10% B, with a flow rate of 0.5 mL min^-1^. Fluorescence detection and quantification were carried out with an excitation wavelength of 230 nm and an emission wavelength of 455 nm [41].

### Statistical analysis

All experiments were independently repeated at least 3 times, each with 3 replicates. One-way ANOVA from SPSS (version 25, Chicago, IL, USA) was used. The differences were considered significant at p ≤ 0.05 using the Duncan test. Graph-Pad version 9 was used for the plot design.

To design cluster heat maps, data files were prepared in comma-separated values (.csv) format and uploaded to the MetaboAnalyst web server (www.metaboanalyst.ca). After processing of uploaded data files by the MetaboAnalyst software, results were downloaded. All data were transformed into Log Normalization to make features more comparable.

## Results

### Impact of medium acidity on growth and paclitaxel yield

**Fig 1:**
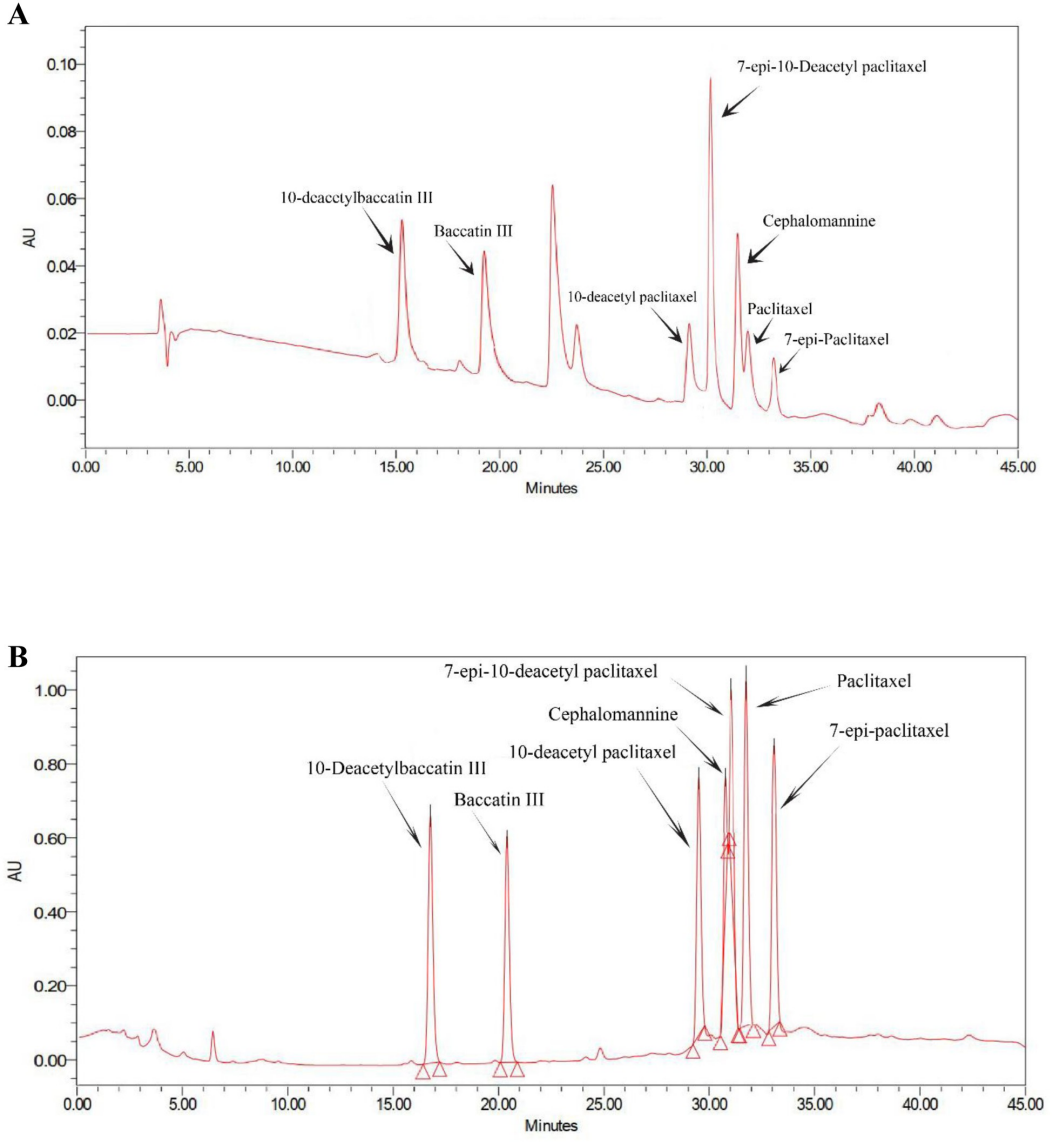
Quantitation of taxanes by HPLC. (A) Taxane profile of *N*. *vitis* in malt extract medium (pH 7.0), (B) profile of standard taxanes.

As mentioned, from the time of isolation of *N*. *vitis*, its ability to produce taxanes was frequently tested by HPLC and LC-MS analysis (Fig 1 and S1 Fig). The taxane profile of the fungus, in comparison with an available mixture of seven taxoids, is shown in Fig 1A and 1B, respectively. 10-Deacetyl baccatin III, baccatin III, 7-epi-10-deacetyl paclitaxel, and cephalomannine were the predominant identified taxoids. 10-Deacetyl paclitaxel and paclitaxel were present in lower quantities, and 7-epi-paclitaxel exhibited the lowest abundance among all detected taxanes. LC-MS analysis confirmed the structural identity of the detected paclitaxel, where the observed [M+H] ion at m/z 853 corresponded to the expected mass of paclitaxel (S1 Fig).

**Fig 2:**
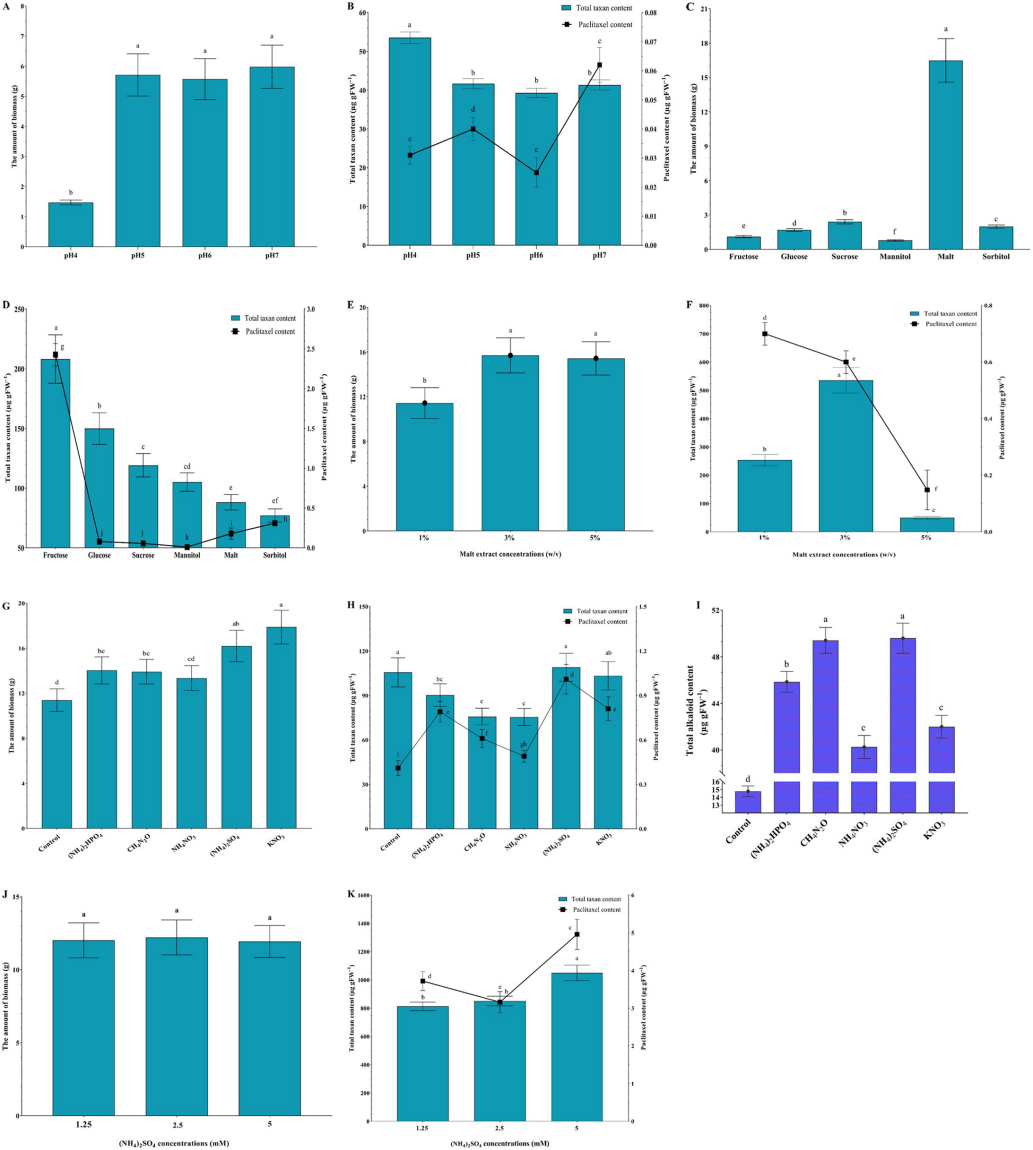
Effects of acidity, carbon, and nitrogen types on growth characteristics, paclitaxel, and total taxane content of *N*. *vitis*. Acidity (A-B), carbon sources (C-D), various concentrations of malt extract (E-F), nitrogen sources (G-H), total alkaloid content (I), and different concentrations of ammonium sulfate (J-K). Data indicate mean ± SD, n = 3. Different letters show significant differences at *p* ≤ 0.05 based on Duncan’s analyses.

The impact of acidity on the growth rate, paclitaxel, and taxane contents of *N*. *vitis* is shown in Fig 2. The lowest growth was observed at the lowest pH (pH 4.0). In contrast, higher pH levels from 5.0 to 7.0 outstandingly improved the growth (Fig 2A). Growing of *N*. *vitis* at pH 4.0 resulted in the highest total taxoid but decreased paclitaxel content, compared to other pH values (Fig 2B). Maximum paclitaxel production was achieved at pH 7.0 (Fig 2B). For this reason, further evaluations of carbon and nitrogen sources were conducted at pH 7.0.

### The optimal carbon source

Among different applied carbon sources, malt extract showed a remarkable effect on increasing the growth rate of *N*. *vitis* (Fig 2C). However, the highest total taxane and paclitaxel contents were detected when fructose was used as the carbon source (Fig 2D).

The effect of different concentrations of malt extract on the growth rate of *N*. *vitis* is depicted in Fig 2E. Significant differences in the growth rates of mycelia are not at 3% and 5% malt extract. However, the highest taxane content was observed at 3% malt extract medium, 2–10 folds higher than other concentrations (Fig 2F). Therefore, adding N-containing compounds to a 3% malt extract medium evaluated different nitrogen source types.

### Nitrogen types and concentrations

Application of exogenous nitrogen sources in the form of potassium nitrate remarkably increased the growth of *N*. *vitis* (Fig 2G). However, feeding *N*. *vitis* ammonium sulfate significantly increased the yield of paclitaxel, reaching about 130% of the control (Fig 2H).

Total alkaloid contents of *N*. *vitis* in different nitrogen supplies are shown in Fig 2I. As shown among the different forms of nitrogen sources, growth in urea and ammonium sulfate resulted in the highest total alkaloid, approximately 240% of the control.

Different concentrations of ammonium sulfate had no significant effects on the growth rate of the fungus (Fig 2J). The highest paclitaxel yield was observed at 5 mM (NH_4_)_2_SO_4_, which was significantly higher than other concentrations (Fig 2K).

The data in Table 1 suggests that using a medium with 3% malt extract and 5 mM ammonium sulfate at pH of 7.0 significantly affected free amino acid levels. As shown, the growth of *N*. *vitis* in this medium was accompanied by an outstanding increase of total free amino acid contents, up to 150% of the control (Table 1). Histidine was the most abundant amino acid in *N*. *vitis* extract under normal conditions. Table 1 shows that its content significantly increased in the optimized medium. It is important to mention the study findings that, when compared to the control, the application of (NH_4_)_2_PO_4_ even decreased the overall amount of amino acids and many amino acids, including asparagine, glutamine, and especially histidine.

**Table 1:** Detected amino acids in *N*. *vitis* in different nitrogen sources. Values are means of three replications ± standard deviation.

	Asparagine	Glutamine	Serine	Histidine	Arginine	Tyrosine	Methionine	Phenylalanine	Isoleucine	Leucine	Total content
						μg gFw^-1^					
**Control**	2.0 ± 0.1 d	5.4 ± 0.1 b	2.1 ± 0.1 b	24.0 ± 1.6 d	0.3 ± 0.1 d	0.01 ± 0.01 f	0.7 ± 0.1 c	0.3 ± 0.01 d	V.L	V.L	33.80 ± 1.4 d
**KNO**_**3**_	7.0 ± 0.3 a	2.6 ± 0.1 c	0.1 ± 0.01 d	55.8 ± 2.8 b	0.9 ± 0.2 b	0.7 ± 0.03 a	2.9 ± 0.1 b	4.9 ± 0.14 a	V.L	V.L	74.04 ± 2.04 b
**NaNO**_**3**_	2.7 ± 0.3 c	6.4 ± 0.1 a	0.3 ± 0.02 c	56.4 ± 2.9 b	0.1 ± 0.01 f	0.06 ± 0 e	0.3 ± 0.03 de	0.6 ± 0.01 c	V.L	V.L	66.75 ± 2.99 c
**Urea**	1.1 ± 0.1 d	1.4 ± 0.1 d	0.08 ± 0.01 e	29.1 ± 1.5 c	1.25 ± 0.2 a	0.3 ± 0.02 c	0.5 ± 0.03 c	0.4 ± 0.03 cd	V.L	V.L	34.10 ± 2.66 d
**(NH**_**4**_ **)**_**2**_ **PO**_**4**_	0.6 ± 0.02 e	1.1 ± 0.1 e	3.8 ± 0.1 a	0.45 ± 0.02 e	0.4 ± 0.04 c	0.45 ± 0.03 b	4.1 ± 0.25 a	4.0 ± 0.3 b	V.L	V.L	14.90 ± 1.35 e
**(NH**_**4**_ **)**_**2**_ **SO**_**3**_	3.1 ± 0.2 b	6.4 ± 0.1 a	0.3 ± 0.04 c	72.90 ± 3.5 a	0.2 ± 0.03 e	0.2 ± 0.03 d	0.3 ± 0.02 e	0.4 ± 0.03 d	V.L	V.L	83.80 ± 3.8 a

Different letters show the significant difference with *p* ≤ 0.05 based on Duncan’s analyses. Means followed by the same letter in each column are not significantly different. VL: very low, the content less than 0.1 μg gFW^-1^

**Fig 3:**
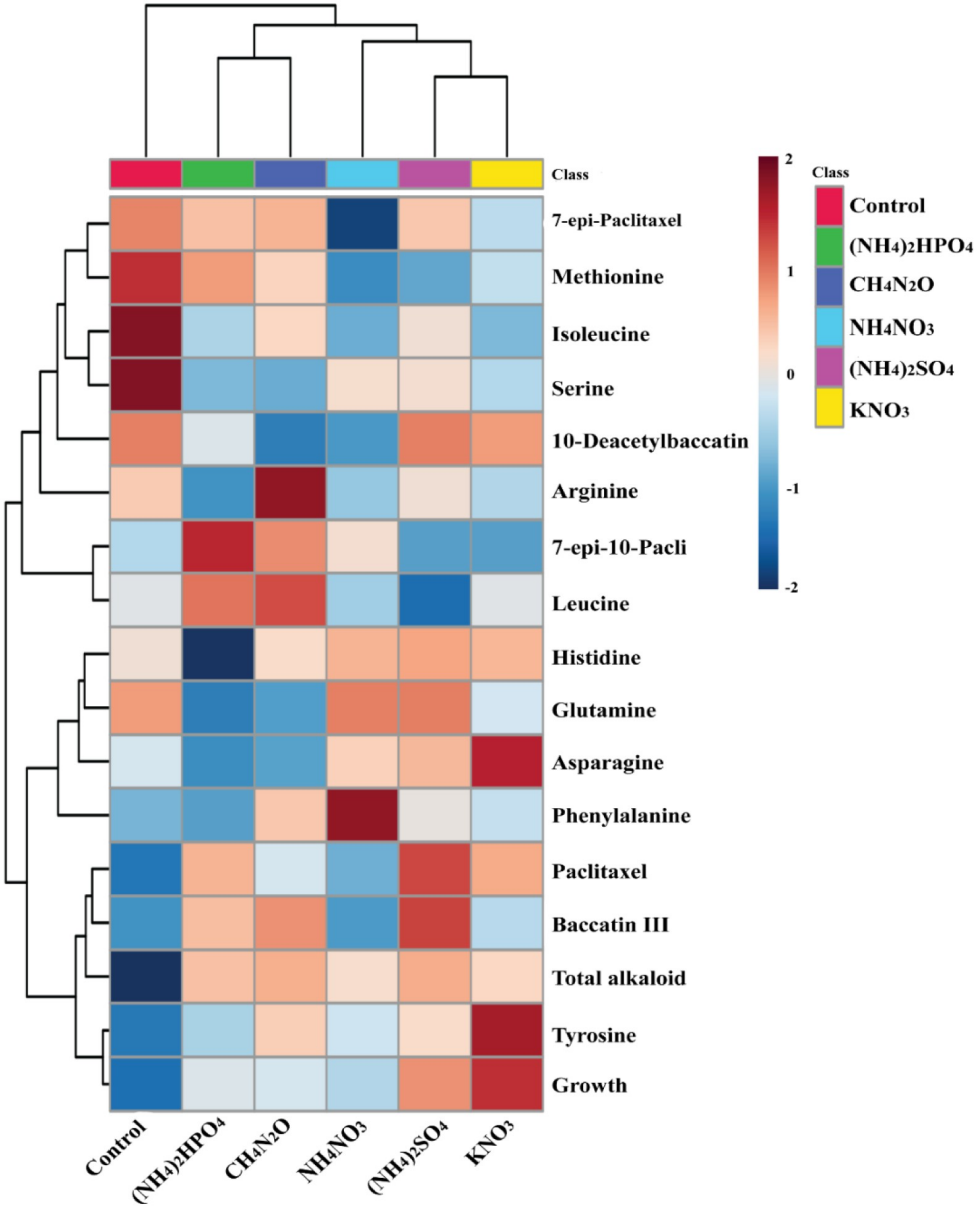
Heat map representation of the correlations between growth rate, amino acids, and taxoid contents of *N*. *vitis* with different nitrogen types. Correlations coefficients were calculated based on Pearson’s method. Red and deep blue indicates positive and negative correlation, respectively.

Correlations between growth, amino acids, total alkaloid, and taxoid contents were illustrated in Fig 3. Positive correlations were observed between ammonium sulfate as a nitrogen source with paclitaxel, 10-deacetyl baccatin III, baccatin III, glutamine, histidine, and total alkaloid contents. Paclitaxel indicated a high correlation to the source of ammonium sulfate. Alkaloids are affected by different carbon sources, but control has a very low correlation with alkaloids and growth. Ammonium phosphate has a low correlation with phenylalanine, but ammonium nitrate can increase its amount compared to other nitrogen sources.

### Optimal growth and taxanes yield in the selected medium

**Fig 4:**
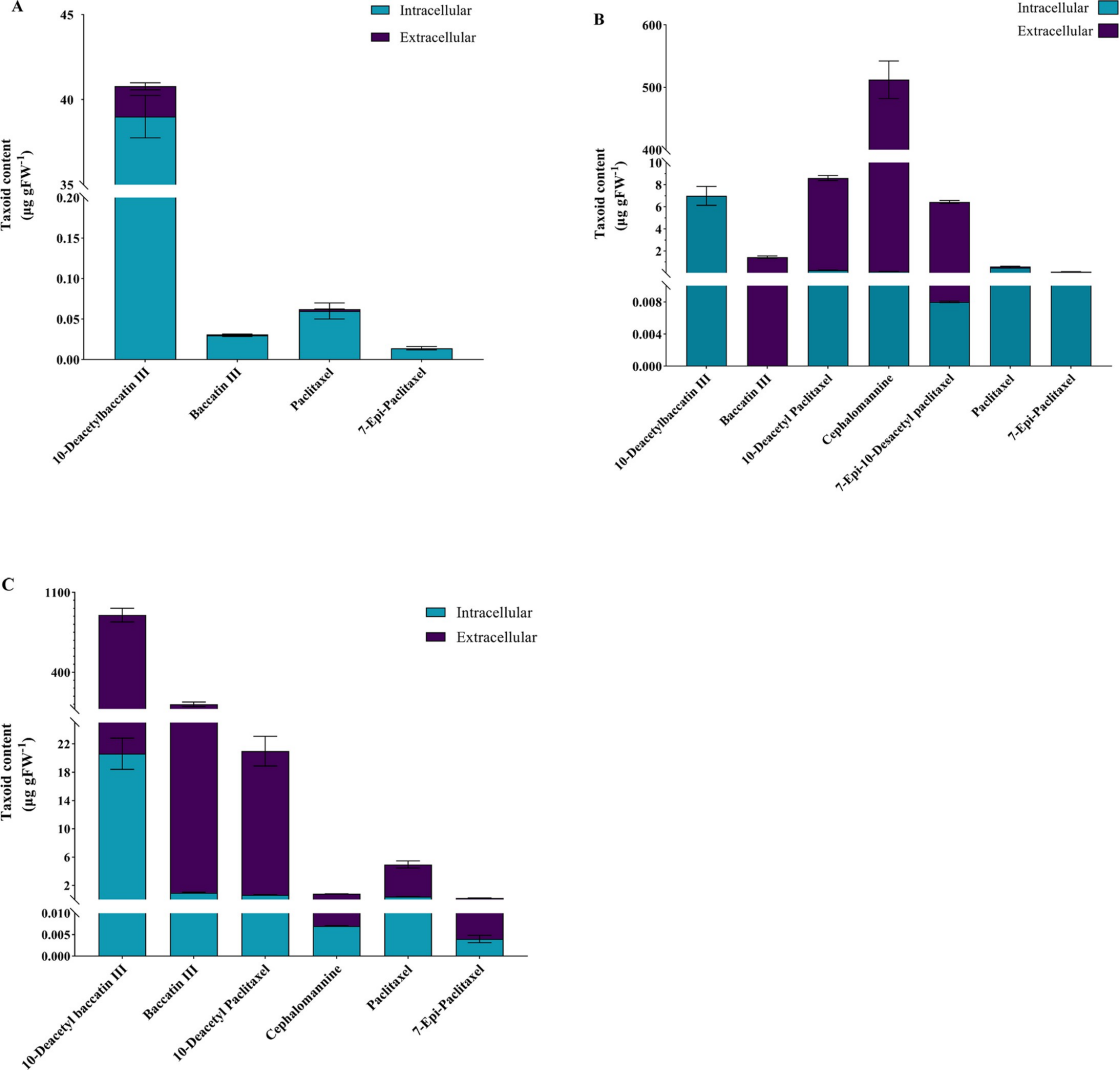
Taxane profiles of *N*. *vitis* along with modification of culture medium. (A) PDB at pH 7.0, (B) malt extract (3% w/v, pH 7.0), (C) the optimized medium containing 3% malt extract and 5 mM ammonium sulfate at pH 7.0.

In the optimized medium, the yield of various taxanes produced by *N*. *vitis* was compared (Fig 4). As shown in Fig 4, different taxoid compounds were produced by *N*. *vitis*.

The taxoid compounds in descending order from 10-deacetyl baccatin III to paclitaxel, baccatin III, and 7-epi paclitaxel were respectively detected in *N*. *vitis* grown in PDB at pH 7.0 (Fig 4A).

The fungus in a solution with 3% malt extract and pH 7.0 produced 7 taxoid compounds, including two newly discovered ones, cephalomannine and 10-deacetyl paclitaxel (Fig 4B). After the addition of 5 mM ammonium sulfate to the medium, it was found that 7-epi 10-deacetyl paclitaxel was not present in the taxanes profile of *N*. *vitis*. The order of taxane yield was determined as follows: 10-deacetyl baccatin III, baccatin III, 10-deacetyl paclitaxel, paclitaxel, cephalomannine, and 7-epi- paclitaxel (as shown in Fig 4C). The profile of taxanes and their quantities were checked in further subcultures of the fungus in the aforesaid medium and the same results were obtained.

## Discussion

The distinctive properties of endophytes, e.g. rapid growth rates, high yield of compounds in a short timeframe, and the growth ability in simple media, highlight their potential for producing significant pharmaceuticals, e.g. paclitaxel. Presence of paclitaxel in endophyte fungi has been detected and quantified by different analytical methods and these fungi have been suggested as highly potent strains on a commercial scale [26, 42, 43]. Paclitaxel biosynthetic genes i.e., 10-deacetylbaccatin III-10-O-acetyl transferase, taxane 13α-hydroxylase and, taxadiene synthase, have also been identified [43–45]. However, the overall architecture of the paclitaxel biosynthetic pathway in endophytes remains unknown [46].

Using chromatographic and spectroscopic methods, followed by PCR-based screening for taxadiene synthase and baccatin III 13-O-3-amino-3-phenylpropanoyl transferase [47], confirmed the molecular blueprint for paclitaxel biosynthesis in *Taxomyces andreanae*. After considerable up-scaling endeavors, however, the commercial isolate brought no confirmation of endophytic paclitaxel production. It has been usually stated that industrial microorganisms tend to gradually reduce or even lose their ability to synthesize molecules of interest in batch cultures. It should be noted however that minimizing the variation of cell growth and production through homogeneous cell line development is a prerequisite for scaling up a culture for production of desired metabolites. Therefore, significant efforts should be accomplished for establishing lines of microorganisms followed by endeavor to their maintenance during the long-term fermentation cycles [48]. Extensive scale-up to commercial production should have considerable consequences for the quality of the product. It is worth noting that, in the present study, *N*. *vitis* has been originally isolated from hazel as a taxane producing endophyte in 2017 and thereafter its ability for taxane production has been frequently examined during 7 years by analytical methods, i.e., HPLC and LC-MS.

There is a wide range of acidity, probably depends on fungi strains, in which fungi can ferment and produce secondary metabolites. Extremely acidic and alkaline media (pH 4.0 and 9.0) drastically reduced the paclitaxel yield of *Aspergillus* [49]. The highest paclitaxel production by *Pestalotiopsis oxyanthi* isolated from *Taxus baccata* was obtained at pH 6.0 [50]. In contrast, pH levels of 6.5 and 5 were respectively found to be the most conducive paclitaxel production by *Taxus* endophytes *Fusarium redolens* and *Paraconiothyrium variabile* [26, 42]. In the present study the best acidity for the maximum paclitaxel production by *N*. *vitis* was pH 7.0.

The precursor availability of fermentation, such as sugars, offers a more convenient environment for microbial growth that promotes metabolite production rates [51]. Recent reports have shown the maximum paclitaxel production by *Alternaria tenuissima* TER995 in glucose, *Aspergillus fumigatus* TXD105, and *Pestalotiopsis oxyanthi* SVJM060 in sucrose containing media [35]. In the present study, sucrose, glucose, sorbitol, malt extract, and mannitol were used to improve the fermentation processes. However, only malt extract showed significant effects on biomass and paclitaxel production by *N*. *vitis*.

Malt extract is mainly composed of maltose, has unique physical properties, is quickly absorbed and broken down into glucose, and provides substantial nutritional benefits. Growth of *N*. *vitis* in malt extract medium was accompanied by an outstanding enhancement of biomass, up to 10 times higher than sucrose, glucose, sorbitol, and mannitol. In the present research, fructose had a notable impact on the paclitaxel content of *N*. *vitis*; however, it did not affect the fungus’s growth compared to other carbon sources. It is interesting to note that fructose has the potential to produce erythrose 4-phosphate via pentose phosphate pathway, which is involved in creating aromatic amino acids and the phenylisoserine side chain of paclitaxel [52]. By calculation, the paclitaxel yield during 1-week growth in fructose medium (2.66 μg g^-1^ FW) is almost equal to the yield of malt extract (2.95 μg g^-1^ FW).

The initial trials in optimization nitrogen type and concentrations revealed that nitrate was superior to ammonium in the increase of paclitaxel production by *Taxus yunnanensis* cells [53]. Ammonium sulfate has been reported as the best nitrogen source for maximal paclitaxel production by *Fusarium solani* Tax-3 [54]. Testing different nitrogen sources on the production of paclitaxel by other endophytes, however, introduced ammonium nitrate as the best form of nitrogen for *Aspergillus fumigatus* TXD105, *Fusarium maire*, and *Epicoccum nigrum* [35, 54, 55].

Based on the present study’s results, ammonium sulfate was an appropriate nitrogen source for producing paclitaxel, while other sources tested in this investigation, i.e., ammonium nitrate and ammonium phosphate, did not yield positive results. The C13-phenylpropanoyl-CoA transferase contributes significantly to paclitaxel biosynthesis by initiating side-chain assembly on baccatin III. It is a typical acyl transferase that is directly involved in the process. In this enzyme, His and Asp side chains residue are thought to be involved in acyl group transfer [56]. Positive correlations were observed between ammonium sulfate, histidine, and the yield of paclitaxel and 10-deacetyl baccatin III.

In addition to C13-phenylpropanoyl-CoA transferase, many acyltransferases are involved in modifying the core taxane skeleton, and these modifications are acylCoA-dependent [57]. Providing precursors for the biosynthesis of CoA, ammonium sulfate-containing medium can accelerate the activity of these enzymes and improve the taxoid yield.

Testing different nitrogen sources on the amino acid content of *Spirulina platensis* introduced urea as the best form of nitrogen [58]. In contrast, nitrogen significantly decreased the amount of amino acids in the root and grains of *Oryza sativa* L. Phosphorus increased the concentrations of acidic and neutral amino acids in the root of *Oryza sativa* L., although it significantly reduced the contents of total amino acids and other amino acids in the grains [59]. It was recently reported that the nitrogen source in the form of NH_4_ had a positive effect on the accumulation of amino acids, especially theaine, glutamate and arginine, and alkaloids [57, 60]. Research suggested that cultivated organisms in NH_4_ demonstrated significantly elevated levels of alkaloids compared to those receiving NO_3_ [60, 61]. As the output of this research, the results highlighted the diverse impacts of different nitrogen sources on the measured parameters. Particularly noteworthy is that the use of ammonium sulfate resulted in elevated levels of alkaloids and amino acids in *N*. *vitis*. The enhancement can be related to several factors, including the pivotal role of ammonium sulfate in gene expression [62], its function as a cofactor for enzymes its and involvement in the synthesis of compounds [63].

A plethora of studies have been conducted to explore the influence of optimizing culture media on the efficacy of taxoids. *Qaio* et al., reported feeding of *Aspergillus aculeatinus* with CuSO4, salicylic acid and sodium acetate improved the paclitaxel yield from 334.92 to 1337.56 μg L^−1^ [64]. *Alternaria alternata* produced the maximum yield of paclitaxel (195.4 μg L^−1^) in a culture media with yeast peptone dextrose broth, at pH 6.0, supplemented with sodium acetate, salicylic acid, and silver nitrate. In the endeavor to enhance the paclitaxel yield, the optimal concentrations of sodium acetate 2.0 g L^-1^, NH_4_NO_3_ 7.8 g L^-1^, and MgSO_4_ 0.68 g L^-1^ were identified for *Fusarium maire*. Following the strain improvement and media optimization, the yield of paclitaxel surged to 225.2 μg L^-1^ [65]. In this investigation and within the optimized medium employed, *N*. *vitis* produced impressive amounts of baccatin III, 10-deacetyl baccatin III, paclitaxel, and 10-deacetyl paclitaxel. The yields were even up to 150, 200, 2.5, and 6 times higher than those in nitrogen free malt extract medium.

## Conclusions

Based upon the findings presented here, cultivation of *N*. *vitis* in a medium containing 3% (w/v) malt extract and 5 mM ammonium sulfate at pH 7.0 resulted in rapid growth and high production of paclitaxel, alkaloids, and amino acids. Interestingly, the growth and paclitaxel production were sustainable and accompanied by the production of other taxanes, including 10-deacetyl baccatin III, baccatin III, 10-deacetyl paclitaxel, cephalomannine, 7-epi 10-deacetyl paclitaxel, and 7- epi-paclitaxel. Keeping the stability of endophyte line and producing high paclitaxel are of the most fundamental needs for commercial targets. In this study, the stability of *N*. *vitis* to produce the highest contents of taxanes after several sub-cultures was clearly confirmed. The results create opportunities to facilitate the production of paclitaxel and its precursors for pharmaceutical industry.

## Supporting information

S1 Fig Chromatogram of extract of *N*. *vitis* analyzed by HPLC-MS. Asterisk refers to paclitaxel in sample (A), ion mass spectrum of paclitaxel in sample (B), and mass spectrum of standard paclitaxel (C).
(DOCX)

## Abbreviations

HPLC: High-performance liquid chromatography
*N. vitis*: *Neopestalotiopsis vitis*
PDA: Potato dextrose agar
PDB: Potato dextrose broth
