# Supplementary figures and images for: Enhancement of paclitaxel production by Neopestalotiopsis vitis via optimization of growth conditions
Source: PLoS One. 2024 Oct 15;19(10):e0309325. doi: 10.1371/journal.pone.0309325 (PMC11478870; doi:10.1371/journal.pone.0309325)

**Fig. 1S**


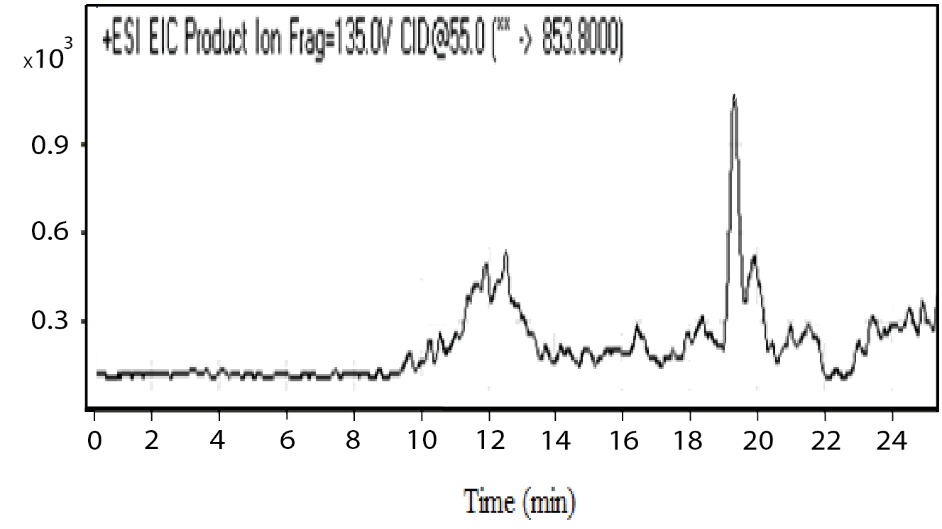


A


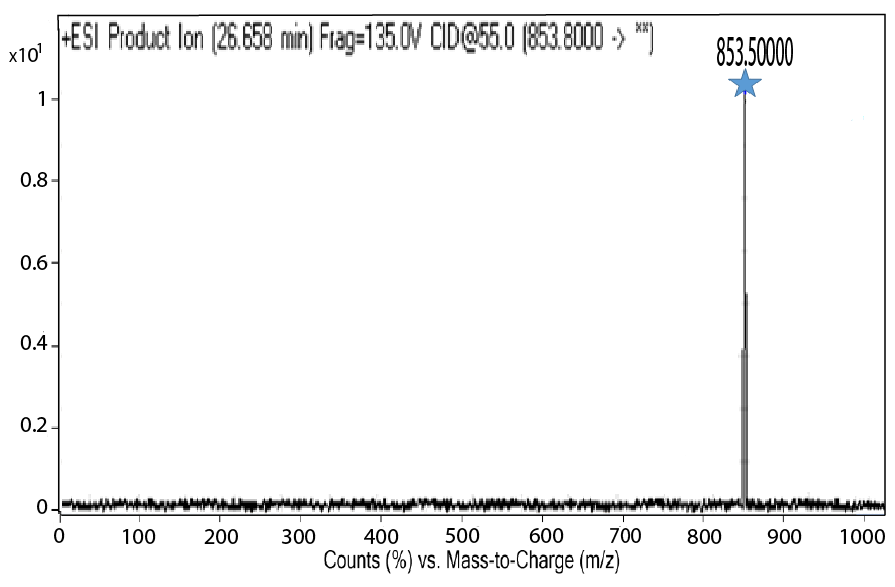


B


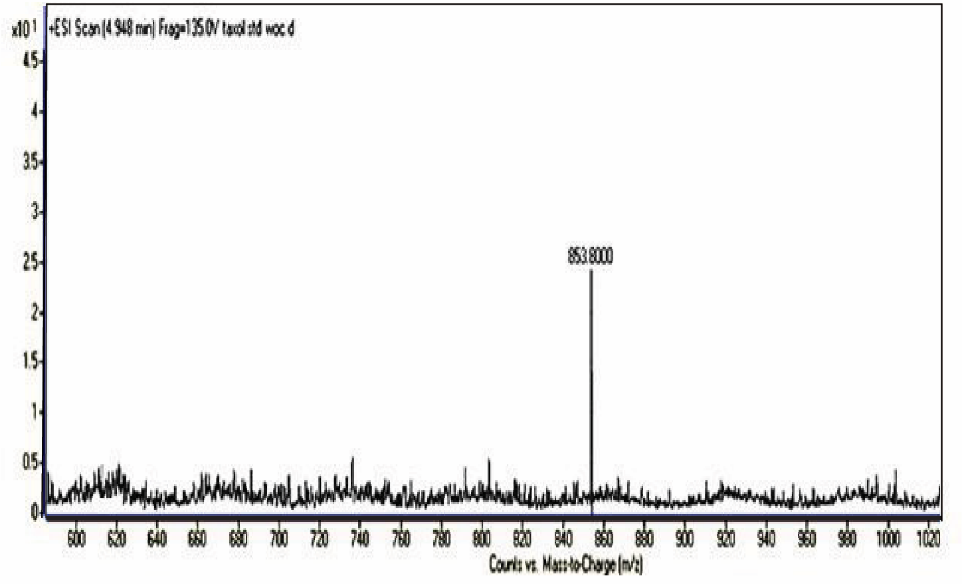


C

Supplement: S1 Fig — Asterisk refers to paclitaxel in sample (A), ion mass spectrum of paclitaxel in sample (B), and mass spectrum of standard paclitaxel (C). (DOCX) [file pone.0309325.s001.docx]
